# Adequate vitamin D status and adiposity contribute to bone health in peripubertal nonobese children

**DOI:** 10.1186/1687-9856-2013-S1-P160

**Published:** 2013-10-03

**Authors:** Jieun Lee, Young Ah Lee, Choong Ho Shin, Sei Won Yang

**Affiliations:** 1 Seoul National University Children’s Hospital, Seoul, Korea

## 

The dietary reference intake (DRI) of vitamin D for Korean children wasreduced from 400IU/day in 2005 to 200IU/day in 2010. We evaluated the risk factors for low vitamin D status and itsrelationships with bone health in peripubertalnonobese children living in Seoul or Gyeonggi-do. One hundred children (9.3 ±1.9 years, 71 prepubertal, 45 boys) participated in the winter (*n* = 38, December through March) and summer (June through September). Bone mineral content (Z_BMC), fat mass (Z_FM), lean mass (Z_LM), and bone mineral density for the total body (Z_TB) and lumbar spine (Z_L1–4) were measured using dual-energy X-ray absorptiometry. Twenty-nine percent of children (47.4% in winter, 17.7% in summer) were vitamin D deficient (25-hydroxyvitaminD level of <20 ng/mL). In winter, low vitamin D intake (*P* = 0.019) and fewer daylight hours (*P* = 0.015) were associated with low 25-hydroxyvitaminD level. The 25-hydroxyvitamin D level correlated positively with Z_BMC (*P* = 0.023), Z_TB (*P* = 0.018), and Z_L1–4 (*P* = 0.043) independently of sex, puberty, Z_FM, Z_LM, physical activity level, and calcium intake. Z_FM correlated independently with Z_BMC (*P*< 0.001), Z_TB (*P* = 0.037), and Z_L1–4 (*P* < 0.001). In conclusion, almost half of peripubertalnonobese children were vitamin D deficient in winter. Considering the beneficial effects of adequate vitamin D status and adiposity on bone health, the current DRI of vitamin D should be upgraded to prevent vitamin D deficiency.

